# Supplementary material for: Gut bacterial diversity and physiological traits of Anastrepha fraterculus Brazilian-1 morphotype males are affected by antibiotic treatment
Source: BMC Microbiol. 2019 Dec 24;19(Suppl 1):283. doi: 10.1186/s12866-019-1645-x (PMC6929401; doi:10.1186/s12866-019-1645-x)
Supplement: Supplementary file 1 — Additional file 1: Figure S1. Alignment of V6-V9 16S rRNA nucleotide sequences (420 bases) obtained from DGGE profiles. Af V6-V9 Seq 1–14 correspond: Band 1 S + P + AB Wild, Band 10 S + P + AB Wild, Band 4 S + P Wild, Band 5 S + P Wild, Band 6 S + P Wild, Band 5 S + AB Wild, Band 3 S Wild, Band 1 S + P + AB Lab, Band 2 S + P + AB Lab, Band 5 S + P Lab, Band 3 S + P Lab, Band 4 S + P Lab, Band 4 S + AB Lab, Band 5 S Lab respectively. [file 12866_2019_1645_MOESM1_ESM.docx]

10 20 30 40 50 60 70 80

....|....|....|....|....|....|....|....|....|....|....|....|....|....|....|....|

**Af V6-V9 Seq 1**  ------------ATGGATTGGTGCCTTCGGGAACTCGAACACAGGTGCTGCATGGCTGTCGTCAGCTCGTGTCGTGAGAT

**Af V6-V9 Seq 2**  -----------ATAGGGTCGGTTCGGCCGGG---TTTCACACAGGTGTTGCATGGCTGTCGTCAGCTCGTGTCGTGAGAT

**Af V6-V9 Seq 3**  --------------GGATTGGTGCCTTCGGGAACTCTGAGACAGGTGCTGCATGGCTGTCGTCAGCTCGTGTTGTGAAAT

**Af V6-V9 Seq 4**  -----------GATGCTTTGGTGCCTTCGGGAACTCTGAGACAGGTGCTGCATGGCTGTCGTCAGCTCGTGTTGTGAAAT

**Af V6-V9 Seq 5**  ------------ATGCTTTGGTGCCTTCGGGAACTCTGAGACAGGTGCTGCATGGCTGTCGTCAGCTCGTGTTGTGAAAT

**Af V6-V9 Seq 6**  ------AAGGGATAGGGTCGGTTCGGCCGGG---TTTCACACAGGTGTTGCATGGCTGTCGTCAGCTCGTGTCGTGAGAT

**Af V6-V9 Seq 7**  -----------GATGCTTTGGTGCCTTCGGGAACCGTGAGACAGGTGCTGCATGGCTGTCGTCAGCTCGTGTTGTGAAAT

**Af V6-V9 Seq 8**  -----------ATAGGGTCGGTTCGGCCGGG---TTTCACACAGGTGTTGCATGGCTGTCGTCAGCTCGTGTCGTGAGAT

**Af V6-V9 Seq 9**  --------------GGATGGGTGCCTTCGGGAACTCGAACACAGGTGCTGCATGGCTGTCGTCAGCTCGTGTCGTGAGAT

**Af V6-V9 Seq 10**  ----------AGATGCTTTGGTGCCTTCGGGAACTCTGAGACAGGTGCTGCATGGCTGTCGTCAGCTCGTGTTGTGAAAT

**Af V6-V9 Seq 11**  ----------AGATGCTTTGGTGCCTTCGGGAACTCTGAGACAGGTGCTGCATGGCTGTCGTCAGCTCGTGTTGTGAAAT

**Af V6-V9 Seq 12**  ----------AGATGCTTTGGTGCCTTCGGGAACTCTGAGACAGGTGCTGCATGGCTGTCGTCAGCTCGTGTTGTGAAAT

**Af V6-V9 Seq 13**  GTTTCGAAGGGATAGGGTCGGTTCGGCCGGG---TTTCACACAGGTGTTGCATGGCTGTCGTCAGCTCGTGTCGTGAGAT

**Af V6-V9 Seq 14**  ----------AGATGGATTGGTGCCTTCGGGAACTCTGAGACAGGTGCTGCATGGCTGTCGTCAGCTCGTGTTGTGAAAT

90 100 110 120 130 140 150 160

....|....|....|....|....|....|....|....|....|....|....|....|....|....|....|....|

**Af V6-V9 Seq 1**  GTTGGGTTAAGTCCCGCAACGAGCGCAACCCTTGTCCTTAGTTGCCAGCACGTAATGGTGGGAACTCTAAGGAGACCGCC

**Af V6-V9 Seq 2**  GTTGGGTTAAGTCCCGCAACGAGCGCAACCCTCATCCTTAGTTACCATCAGGTAATGCTGGGGACTTTAAGGAAACTGCC

**Af V6-V9 Seq 3**  GTTGGGTTAAGTCCCGCAACGAGCGCAACCCTTATCCTTTGTTGCCAGCG-GTTCGGCCGGGAACTCAAAGGAGACTGCC

**Af V6-V9 Seq 4**  GTTGGGTTAAGTCCCGCAACGAGCGCAACCCTTATCCTTTGTTGCCAGCG-GTCCGGCCGGGAACTCAAAGGAGACTGCC

**Af V6-V9 Seq 5**  GTTGGGTTAAGTCCCGCAACGAGCGCAACCCTTATCCTTTGTTGCCAGCG-GTCCGGCCGGGAACTCAAAGGAGACTGCC

**Af V6-V9 Seq 6**  GTTGGGTTAAGTCCCGCAACGAGCGCAACCCTCATCCTTAGTTACCATCAGGTAATGCTGGGGACTTTAAGGAAACTGCC

**Af V6-V9 Seq 7**  GTTGGGTTAAGTCCCGCAACGAGCGCAACCCTTATCCTTTGTTGCCAGCG-GTCCGGCCGGGAACTCAAAGGAGACTGCC

**Af V6-V9 Seq 8**  GTTGGGTTAAGTCCCGCAACGAGCGCAACCCTCATCCTTAGTTACCATCAGGTAATGCTGGGGACTTTAAGGAAACTGCC

**Af V6-V9 Seq 9**  GTTGGGTTAAGTCCCGCAACGAGCGCAACCCTTGTCCTTAGTTGCCAGCACGTAATGGTGGGAACTCTAAGGAGACCGCC

**Af V6-V9 Seq 10**  GTTGGGTTAAGTCCCGCAACGAGCGCAACCCTTATCCTTTGTTGCCAGCG-NTTCGGNCGGGAACTCAAAGGAGACTGCC

**Af V6-V9 Seq 11**  GTTGGGTTAAGTCCCGCAACGAGCGCAACCCTTATCCTTTGTTGCCAGCG-GTTCGGNCGGGAACTCAAAGGAGACTGCC

**Af V6-V9 Seq 12**  GTTGGGTTAAGTCCCGCAACGAGCGCAACCCTTATCCTTTGTTGCCAGCG-NTNCGGNCGGGAACTCAAAGGAGACTGCC

**Af V6-V9 Seq 13**  GTTGGGTTAAGTCCCGCAACGAGCGCAACCCTCATCCTTAGTTACCATCAGGTAATGCTGGGGACTTTAAGGAAACTGCC

**Af V6-V9 Seq 14**  GTTGGGTTAAGTCCCGCAACGAGCGCAACCCTTATCCTTTGTTGCCAGCG-GTTCGGCCGGGAACTCAAAGGAGACTGCC

170 180 190 200 210 220 230 240

....|....|....|....|....|....|....|....|....|....|....|....|....|....|....|....|

**Af V6-V9 Seq 1**  GGTGACAAACCGGAGGAAGGTGGGGATGACGTCAAGTCATCATGGCCCTTACGGCCAGGGCTACACACGTACTACAATGG

**Af V6-V9 Seq 2**  AGTGATAAACTGGAGGAAGGTGGGGATGATGTCAAGTCATCATGGCCCTTATGGAGTGGGCTACACACGTGCTACAATGG

**Af V6-V9 Seq 3**  AGTGATAAACTGGAGGAAGGTGGGGATGACGTCAAGTCATCATGGCCCTTACGAGTAGGGCTACACACGTGCTACAATGG

**Af V6-V9 Seq 4**  AGTGATAAACTGGAGGAAGGTGGGGATGACGTCAAGTCATCATGGCCCTTACGAGTAGGGCTACACACGTGCTACAATGG

**Af V6-V9 Seq 5**  AGTGATAAACTGGAGGAAGGTGGGGATGACGTCAAGTCATCATGGCCCTTACGAGTAGGGCTACACACGTGCTACAATGG

**Af V6-V9 Seq 6**  AGTGATAAACTGGAGGAAGGTGGGGATGATGTCAAGTCATCATGGCCCTTATGGAGTGGGCTACACACGTGCTACAATGG

**Af V6-V9 Seq 7**  AGTGATAAACTGGAGGAAGGTGGGGATGACGTCAAGTCATCATGGCCCTTACGAGTAGGGCTACACACGTGCTACAATGG

**Af V6-V9 Seq 8**  AGTGATAAACTGGAGGAAGGTGGGGATGATGTCAAGTCATCATGGCCCTTATGGAGTGGGCTACACACGTGCTACAATGG

**Af V6-V9 Seq 9**  GGTGACAAACCGGAGGAAGGTGGGGATGACGTCAAGTCATCATGGCCCTTACGGCCAGGGCTACACACGTACTACAATGG

**Af V6-V9 Seq 10**  AGTGATAAACTGGAGGAAGGTGGGGATGACGTCAAGTCATCATGGCCCTTACGAGTAGGGCTACACACGTGCTACAATGG

**Af V6-V9 Seq 11**  AGTGATAAACTGGAGGAAGGTGGGGATGACGTCAAGTCATCATGGCCCTTACGAGTAGGGCTACACACGTGCTACAATGG

**Af V6-V9 Seq 12**  AGTGATAAACTGGAGGAAGGTGGGGATGACGTCAAGTCATCATGGCCCTTACGAGTAGGGCTACACACGTGCTACAATGG

**Af V6-V9 Seq 13**  AGTGATAAACTGGAGGAAGGTGGGGATGATGTCAAGTCATCATGGCCCTTATGGAGTGGGCTACACACGTGCTACAATGG

**Af V6-V9 Seq 14**  AGTGATAAACTGGAGGAAGGTGGGGATGACGTCAAGTCATCATGGCCCTTACGAGTAGGGCTACACACGTGCTACAATGG

250 260 270 280 290 300 310 320

....|....|....|....|....|....|....|....|....|....|....|....|....|....|....|....|

**Af V6-V9 Seq 1**  TAGGGACAGAGGGCTGCAAGCCGGCGACGGTAAGCCAATCCCAGAAACCCTATCTCAGTCCGGATTGGAGTCTGCAACTC

**Af V6-V9 Seq 2**  TGGCTACAATGGGCTGCAAAGTCGCGAGGCTAAGCTAATCCCTTAAAAGCCATCTCAGTTCGGATTGTACTCTGCAACTC

**Af V6-V9 Seq 3**  CATATACAAAGAGAAGCGACCTCGCGAGAGCAAGCGGACCTCATAAAGTATGTCGTAGTCCGGATTGGAGTCTGCAACTC

**Af V6-V9 Seq 4**  CNTATACAAAGAGAAGCGACCTCGCGAGAGCAAGCGGACCTCATAAAGTNNGTCGTAGTCCGGATTGGAGTCTGCAACTC

**Af V6-V9 Seq 5**  CGCATACAAAGAGAAGCGACCTCGCGAGAGCAAGCGGACCTCATAAAGTGCGTCGTAGTCCGGATTGGAGTCTGCAACTC

**Af V6-V9 Seq 6**  TGGCTACAATGGGCTGCAAAGTCGCGAGGCTAAGCTAATCCCTTAAAAGCCATCTCAGTTCGGATTGTACTCTGCAACTC

**Af V6-V9 Seq 7**  CATATACAAAGAGAAGCGACCTCGCGAGAGCAAGCGGACCTCATAAAGTATGTCGTAGTCCGGATTGGAGTCTGCAACTC

**Af V6-V9 Seq 8**  TGGCTACAATGGGCTGCAAAGTCGCGAGGCTAAGCTAATCCCTTAAAAGCCATCTCAGTTCGGATTGTACTCTGCAACTC

**Af V6-V9 Seq 9**  TAGGGACAGAGGGCTGCAAGCCGGCGACGGTAAGCCAATCCCAGAAACCCTATCTCAGTCCGGATTGGAGTCTGCAACTC

**Af V6-V9 Seq 10**  CATATACAAAGAGAAGCGACCTCGCGAGAGCAAGCGGACCTCATAAAGTATGTCGTAGTCCGGATTGGAGTCTGCAACTC

**Af V6-V9 Seq 11**  CATATACAAAGAGAAGCGACCTCGCGAGAGCAAGCGGACCTCATAAAGTATGTCGTAGTCCGGATTGGAGTCTGCAACTC

**Af V6-V9 Seq 12**  CATATACAAAGAGAAGCGACCTCGCGAGAGCAAGCGGACCTCATAAAGTATGTCGTAGTCCGGATTGGAGTCTGCAACTC

**Af V6-V9 Seq 13**  TGGCTACAATGGGCTGCAAAGTCGCGAGGCTAAGCTAATCCCTTAAAAGCCATCTCAGTTCGGATTGTACTCTGCAACTC

**Af V6-V9 Seq 14**  CATATACAAAGAGAAGCGACCTCGCGAGAGCAAGCGGACCTCATAAAGTATGTCGTAGTCCGGATTGGAGTCTGCAACTC

330 340 350 360 370 380 390 400

....|....|....|....|....|....|....|....|....|....|....|....|....|....|....|....|

**Af V6-V9 Seq 1**  GACTCCATGAAGTCGGAATCGCTAGTAATCGCAGATCAGCATTGCTGCGGTGAATACGTTCCCGGGCCTTGTACACACCG

**Af V6-V9 Seq 2**  GAGTGCATGAAGTTGGAATCGCTAGTAATCGTGGATCAGCAC-GCCACGGTGAATACGTTCCCGGGCCTTGTACACACCG

**Af V6-V9 Seq 3**  GACTCCATGAAGTCGGAATCGCTAGTAATCGTAGATCAGAAT-GCTACGGTGAATACGTTCCCGGGCCTTGTACACACCG

**Af V6-V9 Seq 4**  GACTCCATGAAGTCGGAATCGCTAGTAATCGTNGATCAGAAT-GCNACGGTGAATACGTTCCCGGGCCTTGTACACACCG

**Af V6-V9 Seq 5**  GACTCCATGAAGTCGGAATCGCTAGTAATCGTAGATCAGAAT-GCTACGGTGAATACGTTCCCGGGCCTTGTACACACCG

**Af V6-V9 Seq 6**  GAGTGCATGAAGTTGGAATCGCTAGTAATCGTGGATCAGCAC-GCCACGGTGAATACGTTCCCGGGCCTTGTACACACCG

**Af V6-V9 Seq 7**  GACTCCATGAAGTCGGAATCGCTAGTAATCGTGGATCAGAAT-GCCACGGTGAATACGTTCCCGGGCCTTGTACACACCG

**Af V6-V9 Seq 8**  GAGTGCATGAAGTTGGAATCGCTAGTAATCGTGGATCAGCAC-GCCACGGTGAATACGTTCCCGGGCCTTGTACACACCG

**Af V6-V9 Seq 9**  GACTCCATGAAGTCGGAATCGCTAGTAATCGCAGATCAGCATTGCTGCGGTGAATACGTTCCCGGGCCTTGTACACACCG

**Af V6-V9 Seq 10**  GACTCCATGAAGTCGGAATCGCTAGTAATCGTAGATCAGAAT-GCTACGGTGAATACGTTCCCGGGCCTTGTACACACCG

**Af V6-V9 Seq 11**  GACTCCATGAAGTCGGAATCGCTAGTAATCGTAGATCAGAAT-GCTACGGTGAATACGTTCCCGGGCCTTGTACACACCG

**Af V6-V9 Seq 12**  GACTCCATGAAGTCGGAATCGCTAGTAATCGTAGATCAGAAT-GCTACGGTGAATACGTTCCCGGGCCTTGTACACACCG

**Af V6-V9 Seq 13**  GAGTGCATGAAGTTGGAATCGCTAGTAATCGTGGATCAGCAC-GCCACGGTGAATACGTTCCCGGGCCTTGTACACACCG

**Af V6-V9 Seq 14**  GACTCCATGAAGTCGGAATCGCTAGTAATCGTAGATCAGAAT-GCTACGGTGAATACGTTCCCGGGCCTTGTACACACCG

.

**Af V6-V9 Seq 1**  A

**Af V6-V9 Seq 2**  A

**Af V6-V9 Seq 3**  A

**Af V6-V9 Seq 4**  A

**Af V6-V9 Seq 5**  A

**Af V6-V9 Seq 6**  A

**Af V6-V9 Seq 7**  A

**Af V6-V9 Seq 8**  A

**Af V6-V9 Seq 9**  A

**Af V6-V9 Seq 10**  A

**Af V6-V9 Seq 11**  A

**Af V6-V9 Seq 12**  A

**Af V6-V9 Seq 13**  A

**Af V6-V9 Seq 14**  A
